# Phenotyping variants of tumefactive demyelinating lesions according to clinical and radiological features—A case series

**DOI:** 10.3389/fneur.2023.1092373

**Published:** 2023-02-03

**Authors:** Thérèse Boyle, Suran L. Fernando, James Drummond, Ariadna Fontes, John Parratt

**Affiliations:** ^1^Clinical Immunology and Allergy, Royal North Shore Hospital, St Leonards, NSW, Australia; ^2^Immunology Laboratory, Royal North Shore Hospital, St Leonards, NSW, Australia; ^3^Faculty of Medicine and Health, The University of Sydney, Camperdown, NSW, Australia; ^4^Department of Neuroradiology, Royal North Shore Hospital, St Leonards, NSW, Australia; ^5^Department of Neurology, Royal North Shore Hospital, St Leonards, NSW, Australia

**Keywords:** case series, case report, tumefactive demyelinating lesion (TDL), giant demyelinating lesions, Extended Disability Status Scale

## Abstract

**Background:**

Tumefactive demyelinating lesions (TDLs) are defined as lesions >2 cm on MRI of the brain. They are identified in a range of demyelinating diseases including massive demyelination due to Marburg's acute MS, Schilder's Disease, Balo's concentric sclerosis, and Tumefactive MS. Apart from the rare demyelinating variants which are often diagnosed histologically, there are no detailed data to phenotype TDLs.

**Methods:**

We describe the clinical and radiological features of four similar patients with very large TDLs (>4 cm), that are not consistent with the rare demyelinating variants and may represent a distinct phenotype.

**Results:**

All patients presented with hemiplegia and apraxia. The mean age at onset was 37 years with an equal sex distribution. All patients were diagnosed with Tumefactive demyelination based on MRI and CSF analysis, precluding the need for brain biopsy. All responded to potent immunotherapy (including high dose corticosteroids, plasma exchange, rituximab, and/or cyclophosphamide). The mean lag from diagnosis to treatment was 1 day. The median EDSS at presentation was six and recovery to a median EDSS of two occurred over 6 months.

**Conclusion:**

We propose that Tumefactive lesions larger than 4 cm are termed “Giant demyelinating lesions” (GDLs) not only on the basis of size, but a rapid and fulminant demyelinating presentation leading to acute, severe neurological disability that is, nonetheless, responsive to immunotherapy. Further clinical studies are required to ratify this proposed phenotype, establish the immunological profile and best treatment for such patients.

## Introduction

Various terms have been proposed for demyelinating lesions according to their size. Small lesions are described as <3.5 mm (1), whilst large demyelinating lesions are defined as >1 cm (2). MS diagnostic criteria require lesions to be >3 mm in long axis, though those <3 mm with demyelinating characteristics or topography are still considered abnormal (3). The largest lesions, thus far described, are >2 cm in size and historically mimic neoplasms leading to the term tumefactive demyelinating lesions (TDLs). TDLs are detected in at least 1–2/1,000 cases of MS but also occur in acute disseminated encephalomyelitis (ADEM), neuromyelitis optica spectrum disorder (NMO-SD) and the rare variants of MS including Marburg's acute MS, Balo's concentric sclerosis (BCS), and Schilder's disease (4–8).

Marburg's acute MS was first described by Otto Marburg in 1906 and is characterized by large (commonly cerebral hemisphere) lesions with death typically occurring within a year from symptom onset. In 1912, Paul Schilder described a form of demyelinating disease in children and young adults characterized by large cerebral hemisphere lesions with development of new, progressively larger lesions. However, there is debate as to whether this is a unique phenotype or a misdiagnosis of other conditions such as adrenoleukodystrophy (4). Baló's concentric sclerosis (BCS) was named after the Hungarian pathologist József Baló (1895–1979), following his description in 1928 of a demyelinating condition characterized by concentric layers of demyelination and remyelination on MRI or histopathology (9). Balo's lesions can vary in size and occur in the cerebral hemispheres but also basal ganglia, pons, cerebellum, and very infrequently the spinal cord and optic nerves (4).

The demyelinating variants such as Marburg's MS have a particularly aggressive and fulminant course due to their large multifocal lesion volume and extensive axonal transections (10–13). Whilst immunosuppressive treatment has reduced mortality, the morbidity of aggressive Tumefactive demyelination remains high (14, 15). By comparison, smaller TDLs occurring in Tumefactive MS often confer a good prognosis. There is paucity of literature describing the characteristics and prognosis of patients presenting with very large lesions that do not exhibit the phenotypic features of one of the rare demyelinating variants. Furthermore, treatment recommendations for TDLs vary and there are no consensus guidelines (16–18) although it is recognized that Marburg's MS and BCS typically require more aggressive treatment including cyclophosphamide (18–21).

We describe four patients who presented with an acute, monophasic and rapidly evolving neurological syndrome due to very large demyelinating lesions which were >4 cm in size. Their clinical presentations, radiological features and response to treatment are sufficiently homogenous that we propose the term giant demyelinating lesions (GDLs) for the causative abnormalities found on MRI, and describe the outcomes of these remarkable cases.

## Case description and diagnostic assessment

Four patients presented to a tertiary center. One patient presented in 2014, whilst the other three presented between February 2019 and June 2020. All patients suffered from rapidly progressive left hemiparesis with apraxia. None of the patients had acute cognitive changes or encephalopathy. The mean age was 37 years (range 19–55 years) at symptom onset with an equal sex distribution. One patient is Italian ancestrally (migrated from Italy) and the other three patients, Australian. None of the patients were taking regular medication at the time of presentation. The clinical characteristics including medical and family history are summarized in Table 1 and the investigations in Table 2.

**Table 1:** Patient clinical characteristics, diagnosis, treatment, and response.

**Case**	**Sex**	**Age at onset (years)**	**Medical + family history**	**Symptoms + signs at onset**	**Maximum disability (EDSS)**	**Time to treatment from symptom onset (days)**	**Treatment**	**Duration of follow-up (years)**	**Response-latest EDSS**
1	F	19	Personal history of asthma, anxiety, and depression. Nil significant family history reported.	< 24 h left sided numbness with severe weakness and apraxia	6.5	4	Induction: IVMP 1 g for 3 days × 2. 5 × PEX over 2 weeks. Rituximab 1 g × 2; 2 weeks apart, with a slow wean of prednisone. Maintenance: rituximab 6 monthly	2.5	1.5 Lower limb spasticity
2	M	55	Personal history of influenza vaccination 3 weeks prior to symptoms, and recent oral HSV. Nil significant family history reported.	4 days groin pain, followed by acute dysarthria, apraxia and left hemiparesis	8.5	3	Dexamethasone 4 mg bd, levetiracetam 500 mg bd. Broad spectrum antibiotics and acyclovir. Induction: IVIg × 3 days (total 2 g/kg), 1 g IVMP × 5. Rituximab (1 g × 2, 2 weeks apart). Oral prednisone weaning from 1 mg/kg/d over 3 months.	3.5	1 Complete response(fatigue)
3	M	40	Nil	24 h of progressive left sided hemiplegia and apraxia	6.5	2	Induction: PEX × 5 days 1 g IVMP × 5 days. Rituximab 2 × 1 g 2 weeks apart. Weaning prednisone from 50 mg. 2 g/kg IVIg over 3 days, further 5 days of 1 g IVMP followed by oral prednisone weaning from 50 mg. 800 mg/m^2^ cyclophosphamide. Maintenance: Ocrelizumab 6 monthly.	4	2 Mild left upper limb ataxia
4	F	32	Nil prior medical history. Family history of anti-phospholipid syndrome	2 weeks of progressive, severe hemiplegia (leg more affected than arm)	6.5	7	Dexamethasone 4 mg qid, levetiracetam 500 mg bd. Induction: PEX × 5, 1 g IVMP, rituximab 1 g × 2, 2 weeks apart.	8	2 Left lower limb spasticity and spastic gait

IVMP, pulsed intravenous methylprednisolone; PEX, plasma exchange.

**Table 2:** Patient investigations.

**Case**	**Blood test results**	**CSF analysis**	**MRI features at onset**	**Maximum lesion size (mm)**
1	IS negative AIS negative	Cell count: Polymorphs < 1 × 10^6^, mononuclear cells 4 × 10^6^/L, protein 0.19 g/L, intrathecal OCBs, ACSF negative	Giant T2/FLAIR hyperintense right sided periventricular lesion with a maximum dimension of the dominant lesion measuring up to 72 mm. Internal diffusion restriction on DWI and intralesional punctate foci of enhancement on post contrast imaging. There was evidence of one further periventricular T2 FLAIR hyperintense lesion adjacent to the contralateral left occipital horn with similar MRI features including internal diffusion restriction that developed 3 weeks later.	72
2	IS negative AIS negative IGRA indeterminate	CSF 1 (day 2): Cell count: Polymorphs 701 × 10^6^/L, mononuclear cells 17 × 10^6^/L, others 11 × 10^6^/L, protein 1.01 g/l. CSF 2 (day 11): Cell count: Polymorphs < 1 × 10^6^/L, mononuclears nil, others nil, protein 0.53 g/L, absent OCB's, ACSF negative	Solitary, large T2 FLAIR hyperintense lesion centered on the right posterior frontal lobe white matter extending to the ventricular margin with no significant mass effect. Patchy internal diffusion restriction with subtle restricting peripheral margin on ADC. There was interrupted “ring like” peripheral enhancement on the initial scan on post contract T1 imaging.	66
3	IS negative AIS negative IGRA indeterminate	Cell count: Polymorphs 1 × 10^6^/L mononuclear cells 12 × 10^6^, protein 0.41 g/L, intrathecal OCBs, ACSF negative	Solitary 30 mm T2/FLAIR hyperintense lesion involving the right periventricular/peri-atrial white matter. This lesion demonstrated no enhancement or mass effect on the initial MRI however there was marked diffusion restriction seen involving the posterior aspect of this TW FLAIR hyperintesne lesion Sequential development of interrupted “ring-like” enhancement over time (see Figure 1).	47
4	IS negative AIS negative	Cell count: Polymorphs nil × 10^6^/L, mononuclears 1 × 10^6^/L, protein 0.36 g/L, matching OCBs, ACSF negative	Large *T2*/FLAIR hyperintense lesion extending from the right lateral ventricular margin to the cortex of the right posterior *front*al lobe. This dominant lesion involved the posterior body of the corpus callosum. On initial imaging there was wispy associated linear enhancement and regions of both internal and peripheral diffusion restriction. A few non-specific scattered white matter hyperintensities were reported elsewhere with no features to suggest longstanding/disseminated demyelination.	54

IGRA, interferon gamma release assay; IS, infective screen (HIV, hepatitis B and C, syphilis serology, IGRA); AIS, autoimmune screen: (ANA, ENA, ANCA, C3, C4, RF, anti-cardiolipin antibodies, anti-beta-2-glycoprotein antibodies, lupus anticoagulant, ACE); ACSF, additional cerebrospinal fluid analysis: (anti-AQP4 antibodies and JC virus DNA).

**Figure 1:**
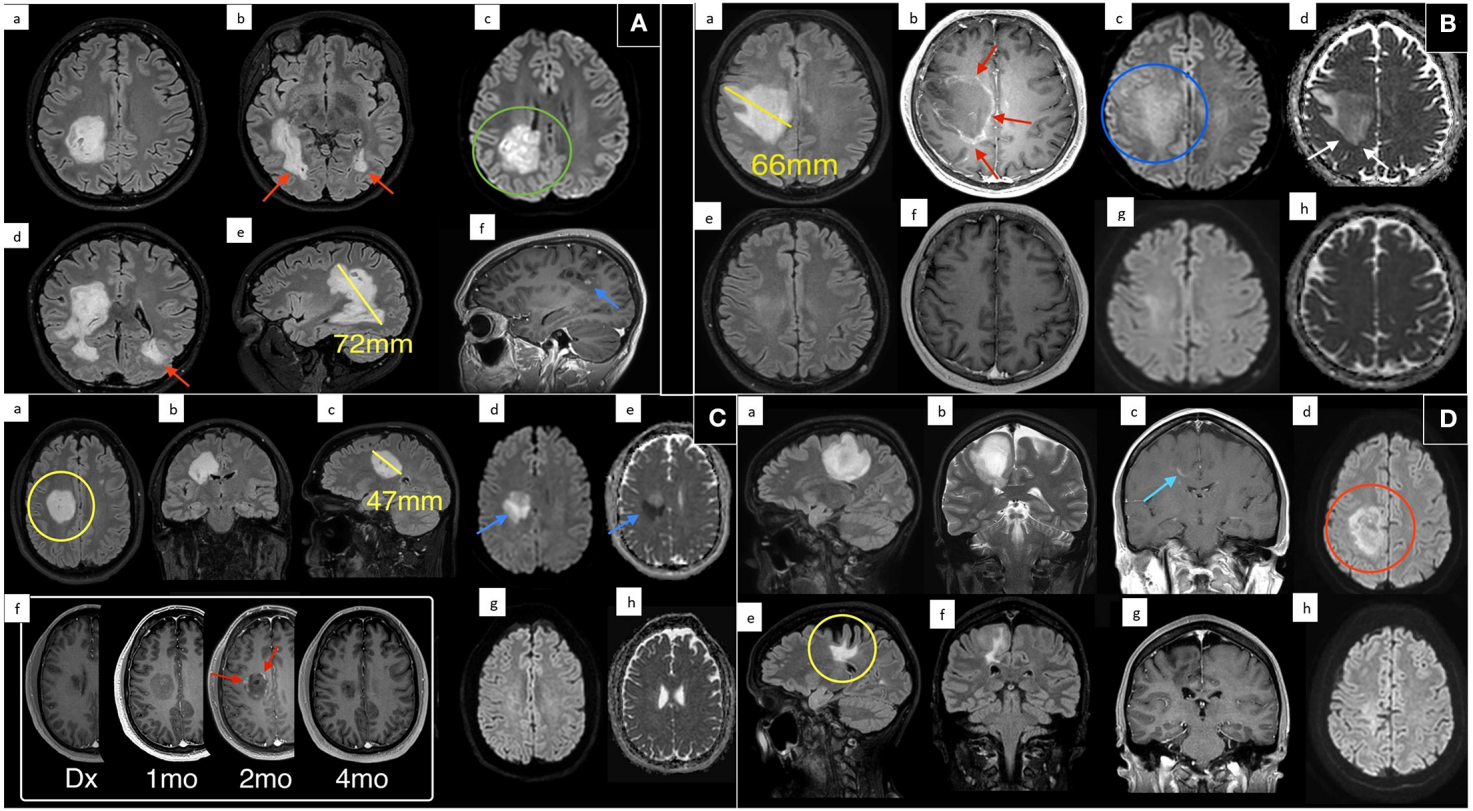
Magnetic resonance imaging of each patient at presentation and various treatment time points. **(A)** Case 1: MRI following presentation demonstrates a giant hyperintense confluent right sided periventricular lesion on axial T2 FLAIR (a/b), with coronal oblique T2 FLAIR reconstruction (d), and sagittal oblique (e) reconstruction. Maximum dimension of the dominant lesion measuring up to 72 mm on sagittal imaging. Evidence of internal diffusion hyperintensity (green circle) on axial DWI (c) and internal punctate foci of enhancement (blue arrow) on sagittal T1 post contrast (f). There was evidence of further periventricular T2 FLAIR hyperintense lesion adjacent to the contralateral left occipital horn (red arrows). **(B)** Case 2: MRI at presentation (a–d) and 4 months follow up (e–h). Scan at diagnosis demonstrates a large solitary T2 FLAIR (a) hyperintense lesion centered on the right frontal white matter with no significant mass effect. This lesion measured up to 66 mm with peripheral enhancement (red arrows) on post contrast T1 imaging (b) with patchy internal diffusion restriction (blue circle) on DWI (c), and subtle peripheral restricting peripheral margin (white arrow) on ADC (d). Near complete resolution on follow up imaging with minimal residual T2 FLAIR hyperintensity (e). **(C)** Case 3: MRI at presentation (a–e), multiple follow up times points post contrast T1 imaging (f), and 4 month follow up DWI (g) and ADC (h). On multiplanar T2 FLAIR (a–c) there is a large periventricular lesion (yellow circle) measuring up to 47 mm with marked associated diffusion restriction (blue arrows) on DWI (d) and ADC (e). No enhancement was demonstrated on scan at diagnosis (c) however the patient progressively developed classic interrupted peripheral enhancement (red arrows). On delayed 4 month follow up scan enhancement and diffusion restriction had resolved (g/h). **(D)** Case 4: MRI at presentation with sagittal T2 FLAIR (a), coronal T2 (b), coronal T1 post contrast (c), and DWI (d) demonstrates a large T2/FLAIR hyperintensity extending from the periventricular margin to the cortex of the right posterior frontal lobe. There was wispy associated linear enhancement (blue arrow) at diagnosis on post contrast T1 imaging (c) and regions of internal and peripheral diffusion restriction (red circle) on DWI (d). Six year follow up scan (e–h) shows marked reduction in FLAIR signal, some associated cortical atrophy and volume loss (yellow circle) and resolution of previous enhancement and diffusion restriction.

All the patients were assessed with CT and CT angiography followed by serial MRI scans (multiple pre and post gadolinium sequences including FLAIR and SWI, Table 2). There was no evidence of hemorrhagic demyelination or vasculitis on any scan. MRI brain demonstrated TDLs in all cases (Figures 1A–D), and the mean size of the lesions at maximum was 59.75 mm (Table 2). Spinal demyelination was absent. All of the lesions demonstrated common radiological features; they were centered on white matter, extended from the lateral ventricular margin to the cerebral cortex in the frontal and parietal lobes and had minimal mass effect. All lesions demonstrated T2/FLAIR hyperintensity with T1 hypointensity and variable patterns of diffusion restriction and gadolinium enhancement (Figure 1). One patient exhibited a Balo-like lesion with a subsequent second much smaller lesion in the contralateral hemisphere (Figure 1A). There was substantial radiological improvement in all cases, with case 1 and 2 showing near-complete resolution of the GDLs (Figures 1A, B).

The CSF was generally bland with a mononuclear pleocytosis in only one case. Two patients had intrathecal synthesis of oligoclonal bands, one had matched serum and CSF bands and the CSF was normal in Case 2 (Table 2). All patients were negative for anti-aquaporin-4, anti-myelin oligodendrocyte glycoprotein (MOG) and anti-neuronal antibodies in CSF and/or serum.

Based on the severity of the clinical presentation and results of investigations, treatment was prioritized in all cases. Neurosurgical opinion was sought for case 3 though biopsy was deferred due to subsequent rapid improvement following additional treatment. Other diagnoses were considered but high grade Glioma was excluded based on radiological features (CT and MRI) and CSF abnormalities in most cases, neurosarcoidosis principally on MRI, infection from the incongruent clinical presentation (absence of fever and obtundation) and bland CSF, and lymphoma principally on MRI findings and in one patient of concern; hypometabolism of the lesions on FDG-PET.

**Figure 2:**
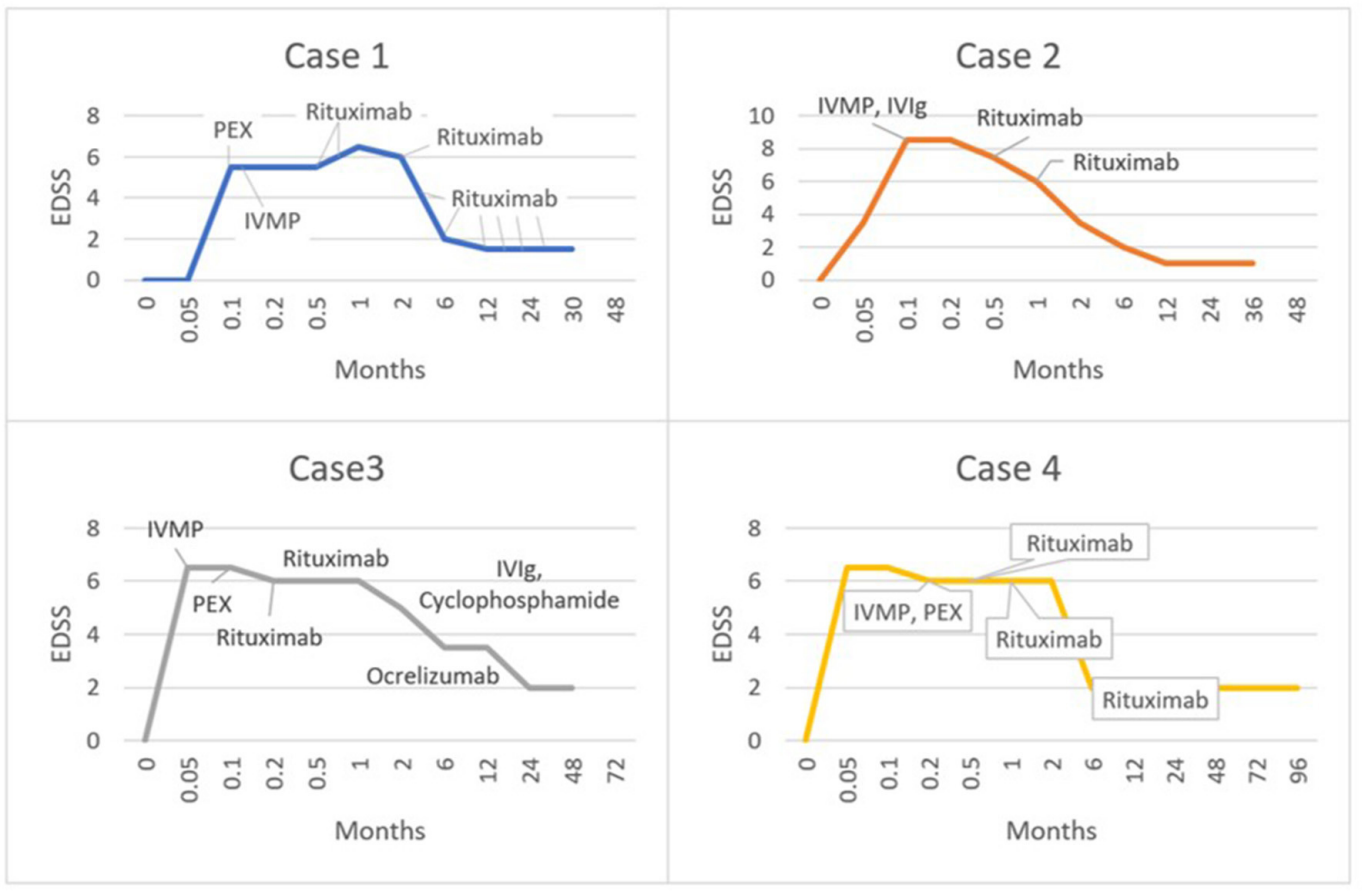
Graphs illustrating the clinical response of each patient according to timing and nature of treatment, as represented by the Extended Disability Status Scale (EDSS).

All patients were treated with intravenous methylprednisolone (IVMP) and plasma exchange, followed by rituximab. One patient had cyclophosphamide as an adjunctive treatment (outlined in Table 1; Figure 2). Patients also received supportive care with physiotherapy to aid recovery. The clinical response to treatment, as measured by the Extended Disability Status Scale (EDSS) is shown in Figure 2. The median maximum EDSS was 6.5 and severe neurological disability developed quickly reaching this point at a mean of 9 days after onset. However, all patients recovered steadily with aggressive treatment reaching a median EDSS of 2 (after a mean follow-up duration of 4.5 years). Patients 1 and 3 required maintenance treatment, whilst patients 2 and 4 were treated with induction treatment alone. All patients were monitored in the outpatient setting with clinical assessment including EDSS and repeat MRI every 3–6 months. All patients were adherent with treatment and incurred no adverse effects on clinical assessment and regular laboratory tests. The diagnosis for all patients remains unspecified Tumefactive demyelination albeit patient 1 had radiological features of BCS and patient 2 had a precipitant and onset similar to ADEM. Patient 3 likely has Tumefactive MS, though remains in remission following an unusual monophasic presentation with a TDL at the onset. All patients exhibited very large lesions, with similar characteristics that suggest that these cases could be termed GDLs.

## Discussion

Tumefactive demyelinating lesions are identified principally on MRI and specific diagnostic criteria have been proposed, but not validated or widely implemented (22). The radiographic features that are suggestive of demyelination include incomplete rim enhancement, a peripheral margin of diffusion restriction, absence of mass effect and generally, lack of cortical involvement (23, 24). MS is one of the causes while other pathologies including infection, autoimmune diseases and malignancy can be reasonably excluded using the clinical history, autoimmune serology, microbiology, CSF-analysis and additional imaging (11, 14, 25–30). It is seldom necessary to undertake a brain biopsy to facilitate the diagnosis of Tumefactive demyelination (13, 31).

Distinguishing lesions >4 cm as GDLs identifies a group of patients at high risk of neurological morbidity. While GDLs clearly share many features of TDLs it is our view that the distinctions of much lower incidence (in our experience), greater size and severity, speed of evolution and cortical involvement are sufficient to categorize this group as a particular and rare entity (32). Based on clinical and radiological data, GDLs grow rapidly from the ventricular surface and the leading edge of the lesion does not stop until the cerebral cortex is involved, sometimes to the level of the pia. They are quickly destructive as shown by rapid clinical deterioration, the development of chronic T1 hypointense changes in the core of the lesion, and lasting, focal cerebral atrophy. The diagnosis in each case here was made based on features suggestive of demyelination radiologically and CSF findings, particularly the presence of oligoclonal bands (OCBs) (27, 33).

It can be reasonably assumed, in the absence of histopathology, that the lesions described in our case series are demyelinating based on two main findings: the rapid evolution of neurological deficits implying conduction block and the radiological similarities to TDLs that have been biopsied in some series (12, 34). One of the patients met criteria for multiple sclerosis at presentation, with a couple of trivial demyelinating lesions elsewhere implying a common mechanism with that disease, but another patient exhibited MRI features of Balo's phenomenon, whilst a third might have been considered an ADEM variant because of preceding viral infection and vaccination, with remarkable recovery. However, the Balo's type GDL responded to plasma exchange and rituximab which is inconsistent with the proposed mechanism of that condition; a primary loss or destruction of oligodendrocytes in the absence of humoral immunological factors (4, 35), and ADEM is characterized by multifocal small perivenous demyelinating lesions (36, 37). Indeed, the lesions described in our case series do not fit well with any previously described demyelinating condition (14, 27).

The pathogenesis of TDLs is complex and probably involves multiple genetic and immunological mechanisms (15, 38, 39). It is interesting that T-cell dysregulation as seen in HIV infection, nivolumab and natalizumab treatment may be the substrate for TDLs in some cases, and similarly T-cell subset shifting through treatment with fingolimod (17, 18, 40, 41). This might lead to dysregulation of B-cell and plasma cell immune responses by altering the immunoregulatory T-cell phenotype. Occasional reports have identified IgG and complement deposition in TDLs (42–44) and a number of reports citing the therapeutic effects of plasma exchange and rituximab imply B-cell and IgG-mediated mechanisms are also important (17, 27, 40, 41). Proven antibody mediated diseases such as NMO-SD are associated with TDLs (4). Humoral abnormalities were shown in all but one of our patients. The absence of OCBs in case 2 may reflect the timing of collection during the disease course, and does not exclude an antibody mediated process with a consistent clinical-MRI phenotype (45). Notably, the patient responded to PEX and Rituximab. The pathogenesis of ADEM is also unclear though it is typically triggered by an infection or vaccination with subsequent molecular mimicry as the proposed mechanism of disease (46).

By inference as a subgroup or variant of TDLs, GDLs exhibit features consistent with a humoral pathogenesis, and all the patients responded in some way to plasma exchange or intravenous immunoglobulin. Of course, the treatment was not exclusive and intravenous methylprednisolone would have reduced granulocyte functions and cytokine release within the lesion (47). Moreover, one patient required cyclophosphamide as an adjunctive treatment to rituximab, attenuating T-cell responses as well as diminishing antigen presentation from B-cell ablation (16, 48). In case studies PEX (18, 40, 41), rituximab and cyclophosphamide are beneficial and safe (16–18, 49, 50). Mitoxantrone and natalizumab are variably effective (18), but the latter can cause TDLs in NMO-SD (19).

We acknowledge that TDLs are quite variable in their etiology and pathological mechanisms and recent work indicates that the treatment algorithm should be adjusted for phenotypic differences and age. For example, Tzanetakos et al proposed treatment is tailored to patients depending on whether they exhibit Balo's phenomenon (19, 20). There have been no clinical trials to date or formal treatment guidelines for TDLs and, by and large, an escalation approach is recommended from high dose corticosteroids to PEX followed by rituximab and/or cyclophosphamide according to response, with consideration of brain biopsy for refractory cases (19). In our series, time and brain were conserved with the inception of corticosteroid therapy and plasma exchange whilst the investigations were continuing to complete the diagnosis. Patients were treated quickly and aggressively with the intention of maximizing the effect of rituximab (an anti-CD20 B-cell depleting monoclonal antibody) through a leaky blood brain barrier early in the course of the disease. We think the rapidity of B-cell suppression may have influenced the disability outcomes in these patients which were excellent.

Patients with TDLs have a variable prognosis, and one-third develop MS over a period of up to 5 years (4). There is lack of consensus as to the maintenance therapy for TDLs (10, 18). Adverse prognostic factors include lesion size >5 cm, infiltrating lesions and older age (10, 11, 51). Patients with established MS who develop TDLs, or who subsequently meet criteria should receive conventional recommended MS treatment but with caution when switching from natalizumab or fingolimod and where the pathogenesis is uncertain, B-lymphocyte suppression may have broader efficacy (35). Those with TDLs who do not meet criteria for MS should be treated according to the severity of the disease and treatment response. To this point, two patients in our series continue B-cell immunosuppressive treatment and two have ceased therapy without any relapses. None of the patients have developed new lesions over a prolonged period of follow up.

This is the first report comprising patients with very large demyelinating lesions that do not fit classical descriptions of multiple sclerosis or the rare demyelinating variants. We propose these patients have a distinct phenotype and are unified by the presence of giant demyelinating lesions (GDLs). We suggest using this criterion, with other clinical and laboratory data, to lead to rapid diagnosis and treatment.

However, we acknowledge the significant limitations of a case series design and the small sample size. Further collective work is required to better elucidate the outcomes and response to treatment in patients with GDLs. Brain biopsies were not performed in our cohort of patients as their disease responded to treatment and because imaging and ancillary tests were consistent with a demyelinating process. Whilst this reduces iatrogenic risk and shortens the time to maximum potency treatment, histopathological study would elucidate the nature of the inflammatory infiltrate allowing for more targeted or tailored treatment regimens. In time, perhaps genetic testing may also have utility in this rare condition (32, 52).

Finally, characterizing specific groups of patients with tumefactive demyelinating lesions may facilitate a search for biomarkers and pathomechanisms similar to the successes recently realized in autoimmune encephalitis (53, 54) and Myelin Oligodendrocyte Glycoprotein Antibody Disease (55, 56). Our clinical and treatment data imply GDLs are mediated, at least in part, by humoral mechanisms and further efforts should be made in this field to facilitate precision phenotyping and accurate treatment (57).

## Patient perspective

All four patients expressed their initial concern regarding possible irreversibility of this unusual condition and therefore relief from effective and well-tolerated treatment with functional recovery and ability to return to work. All patients are highly supportive of ongoing research on this condition.

## Data availability statement

The original contributions presented in the study are included in the article/supplementary material, further inquiries can be directed to the corresponding author.

## Ethics statement

Written informed consent was obtained from the individual(s) for the publication of any potentially identifiable images or data included in this article.

## Author contributions

JP and SF designed the study. JP, SF, AF, and TB were the primary carers whilst all authors participated in case management. The manuscript was written by TB and all authors commented on versions of the manuscript. All authors read and approved the final manuscript.
